# Abductor muscle function after anterolateral approach in patients with unilateral end-stage hip osteoarthritis undergoing total hip arthroplasty: A prospective study

**DOI:** 10.3389/fsurg.2023.1134355

**Published:** 2023-03-24

**Authors:** Siwadol Wongsak, Chavarat Jarungvittayakon, Kulapat Chulsomlee, Suphaneewan Jaovisidha, Paphon Sa-ngasoongsong

**Affiliations:** ^1^ Department of Orthopedics, Faculty of Medicine Ramathibodi Hospital, Mahidol University, Bangkok, Thailand; ^2^ Department of Diagnostic and Therapeutic Radiology, Ramathibodi Hospital, Mahidol University, Bangkok, Thailand; ^3^ Chakri Naruebodindra Medical Institute, Faculty of Medicine Ramathibodi Hospital, Mahidol University, Bangkok, Thailand

**Keywords:** total hip arthroplasty, hip abductor muscle power, anterolateral approach, gluteus medius muscle, hip osteoarthritis article type: original research article

## Abstract

**Background:**

Total hip arthroplasty (THA) is a successful procedure for treating end-stage hip osteoarthritis (OA). Regarding the surgical approach for THA, the anterolateral (AL) approach, which requires anterior hemimyotomy of the gluteus medius muscle, has shown a long-term favorable outcome. However, to date, complete information related to hip abductor muscle outcomes after the AL approach is unavailable. This study therefore aimed to evaluate the postoperative outcome of patients who undergo THA using the AL approach in terms of hip abductor muscle recovery, pain, function, and muscle healing status.

**Methods:**

Twenty patients diagnosed with unilateral end-stage hip OA underwent cementless THA with the AL approach. All patient procedures were performed by a single surgeon. Preoperative and postoperative data were collected at 2-week, 6-week, 3-month, and 6-month follow-up periods. Hip abductor muscle power was measured *via* handheld dynamometer. The healing of the musculotendinous repair was evaluated with magnetic resonance imaging at 9 months.

**Results:**

After THA, hip abductor muscle power in the operated hip significantly increased as early as 3 months post-procedure when compared with the preoperative value (*p *< 0.05). The other parameters—including pain score, Harris hip score, and WOMAC score—significantly improved as early as 2 weeks post-operation (*p *< 0.05). In all patients, MRI scans showed good healing of the muscle repair site without a gap in the gluteus medius muscle. However, three patients (15%) had some fibrosis and tendon swelling at the repair site.

**Conclusion:**

This study demonstrated that patients with end-stage hip OA could experience significantly improved hip abductor motor function as early as 3 months after undergoing THA with the AL approach. Moreover, despite patients experiencing anterior hemimyotomy of the gluteus medius muscle, no significant complications emerged at the muscle repair site in the AL approach.

## 1. Introduction

Total hip arthroplasty (THA) is one of the most successful procedures in orthopedic surgery (1). THA is an effective method in young and older patients who experienced pain and dysfunction from arthritic hip joints unrelieved by conservative treatment (2, 3). Generally, THA can be performed *via* a variety of surgical approaches, such as the anterolateral (AL), direct anterior (DA), and lateral and posterior approaches (4). Among those, the anterior approach (either AL or DA) has emerged as a preferred method for achieving successful early postoperative outcomes (5, 6).

Concerning the anterior approaches, the AL approach has many advantages, such as good exposure of the acetabulum, implant positioning, leg length correction, and decreased incidence of dislocation (7–10). However, the AL approach has a drawback: the need to perform anterior hemimyotomy of the gluteus medius for better exposure, which requires muscle repair at the end of the operation. This iatrogenic muscle injury results in a concern about postoperative hip abductor weakness and possible complications related to unhealed muscle repair as hip dislocation (11, 12). On the other hand, while the DA approach has recently become a popular minimally evasive approach over the past decade, related complications—such as periprosthetic fracture in the early learning curve (13, 14), high risk of wound complications in obese patients (15), and difficulty in the dysplastic hip (16)—have been frequently reported.

Yet to the best of our knowledge, only a few previous studies have reported on the postoperative hip abductor muscle outcome after using the AL approach (17, 18), and no data is available regarding the hip abductor muscle recovery, the prognosis of abnormal hip function (e.g., Trendelenburg gait), and the status of the gluteus medius muscle healing. Addressing that information gap, we aimed to help better understand the effect of the AL approach in patients with end-stage hip OA undergoing THA in terms of the clinical outcome and the postoperative change of abductor muscles. We therefore conducted a prospective study using patients with unilateral end-stage hip disease and performed THA using the AL approach. The goals were to evaluate the postoperative hip abductor muscle power, clinical outcomes, and continuity of abductor muscles using magnetic resonance imaging (MRI).

## 2. Materials and methods

This study was approved by the Ethical Clearance Committee on Human Rights Related to Research Involving Human Subjects, Faculty of Medicine Ramathibodi Hospital, Mahidol University (Protocol number: ID 10-58-09).

For the study, we recruited 20 patients diagnosed with unilateral end-stage hip OA from Ramathibodi Hospital, Thailand. The patients underwent cementless THA with the AL approach from January 2016 to December 2017. Inclusion criteria were patients diagnosed with end-stage hip OA and unilateral hip disease with positive Trendelenburg gait. Exclusion criteria were patients with a previous surgery, a previous hip joint dislocation, and neuromuscular weakness.

Patients completed a preoperative interview and questionnaire administered by one investigator; the data collected included age, sex, body mass index, pain score, range of motion, gait, abductor muscle power, Harris hip score, and WOMAC score.

**Figure 1:**
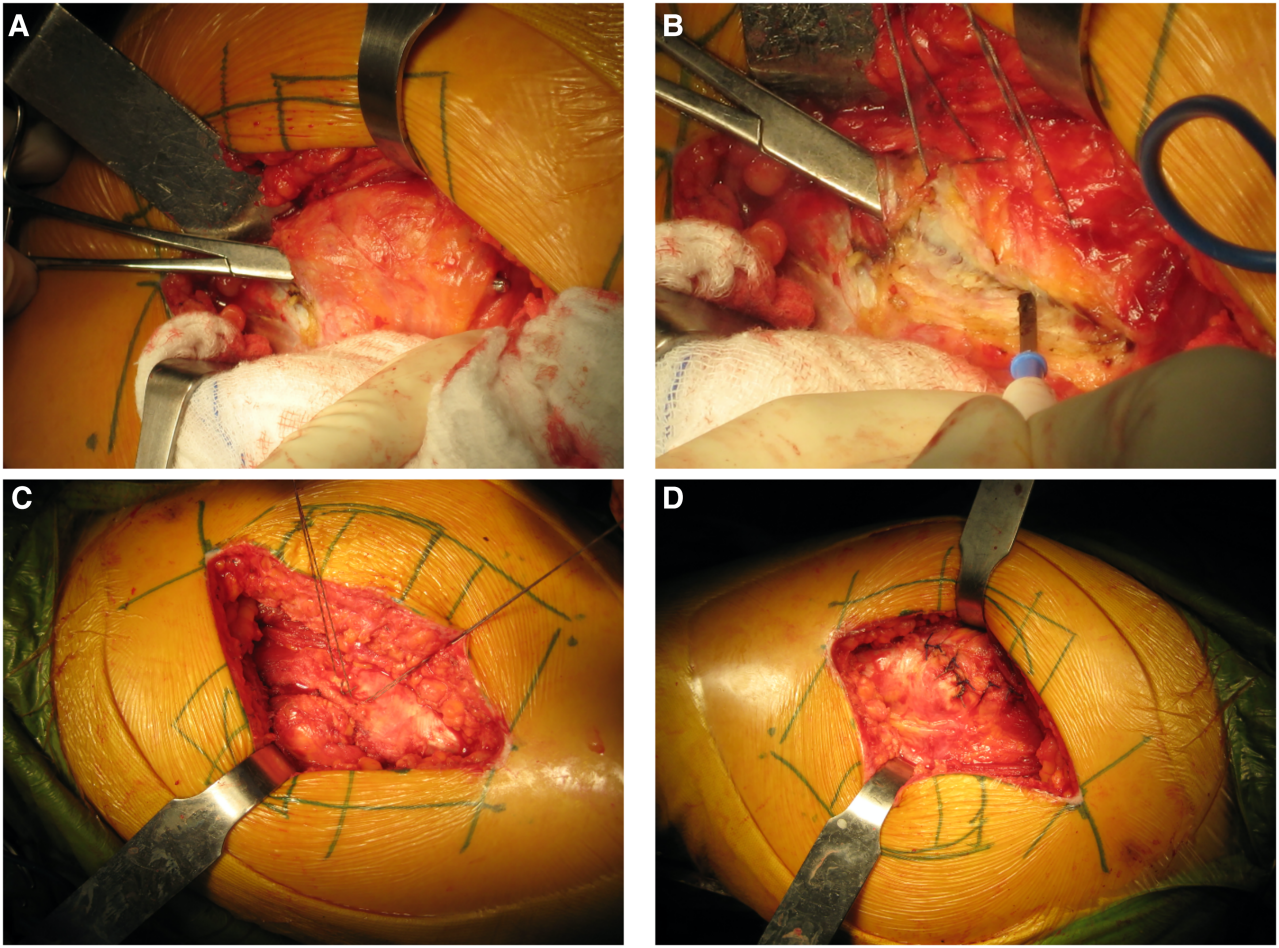
Surgical technique for the AL approach. Following splitting tensor fascia lata, the anterior one-third of the gluteus medius was identified (**A**) and cut (**B**). When the prosthesis was inserted and finally checked for stability, the musculotendinous flap of the gluteus medius and minimus were sutured (**C**) and anatomically repaired (**D**).

After giving informed consent, all patients subsequently underwent an operation performed by a single surgeon who has used the AL approach in more than 1,000 cases (SW). All patient surgeries used cementless implants for both the femoral and acetabular components. The implant position was the preoperative template for achieving the anatomical position using the contralateral side as a reference. For all patients, the AL approach was performed *via* a longitudinal split of the tensor fascia latae and iliotibial band, reflecting anteriorly the anterior one-third of the gluteus medius and gluteus minimus (Figures 1A,B). The acetabular cup was set as 15 ± 10 degrees anteversion and 40 ± 10 degrees inclination according to the Lewinek's safe zone (19). After implantation, the musculotendinous flap was repaired in the anatomical position for both the gluteus medius and the gluteus minimus (Figures 1C,D).

All patients underwent the same rehabilitation protocol starting the day after surgery and then followed the same outpatient protocol. The physiotherapy protocol included gait training, abductor muscle strengthening exercise, and instructions on safe self-administered exercise protocols. Patients were assessed postoperatively at 2-week, 6-week, 3-month, and 6-month intervals. For each patient, we collected the following data at all intervals: pain score (VAS score), range of motion, gait, abductor muscle power, Harris hip score, and WOMAC score. Abductor muscle power was evaluated by a single evaluator based on the newton unit, as measured with the MicroFET 2 (12-0381W) digital handheld dynamometer.

**Figure 2:**
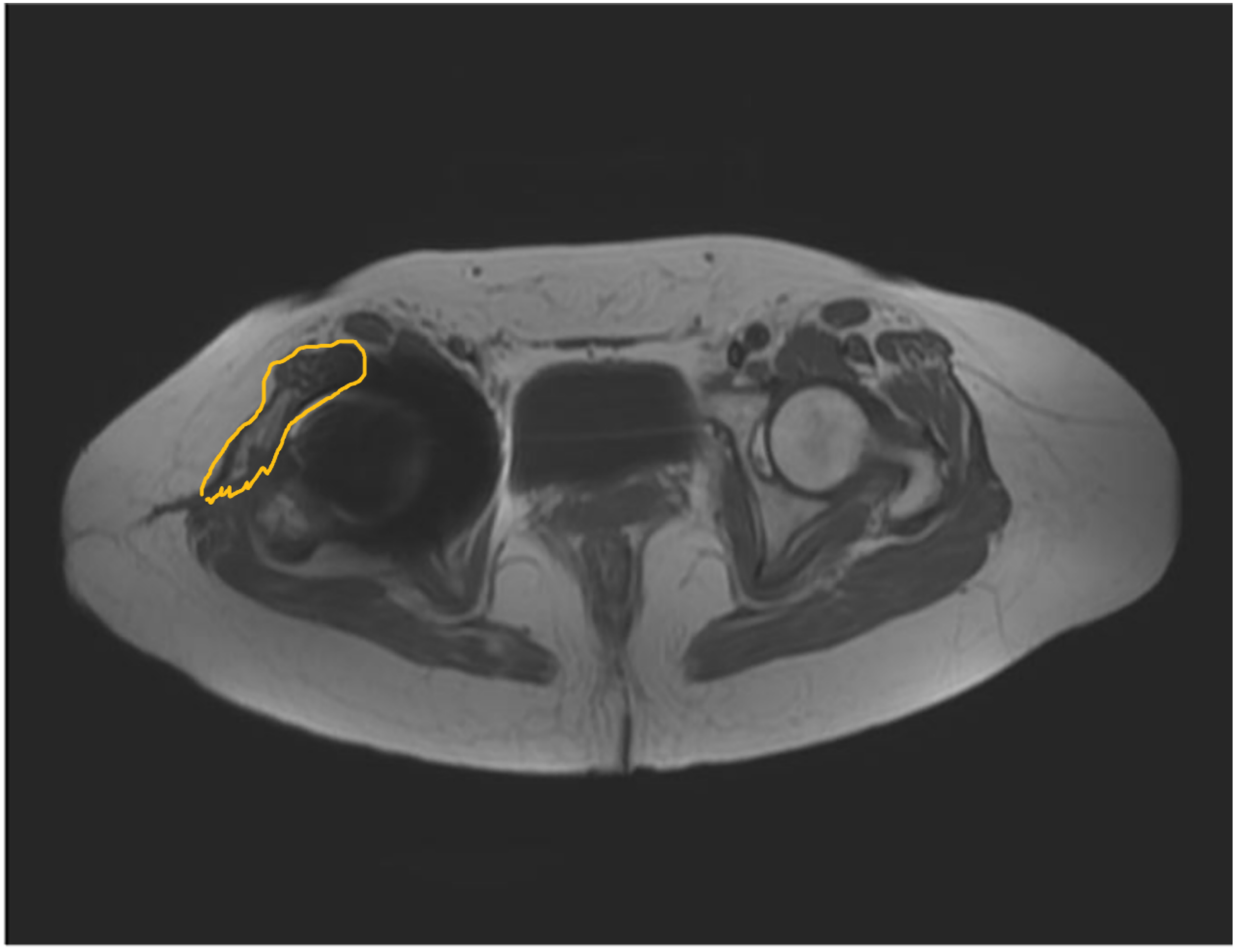
Continuity of the musculotendinous flap (yellow line) was evaluated by magnetic resonance imaging (MRI) at 9-month interval postoperatively.

An MRI was performed postoperatively for all patients at the 9-month interval to evaluate the degeneration of muscle and the continuity of muscle or musculotendinous junction, as described in previous studies (20, 21); all MRIs were performed by an experienced senior musculoskeletal radiologist (SJ). The continuity of the musculotendinous flaps was collected to evaluate the healing of the musculotendinous flap of the gluteus medius after the anterior hemimyotomy was performed and X was repaired intraoperatively (Figure 2).

All statistical analyses were calculated using MedCalc Statistical Software version 15.8 (MedCalc Software bvbv, Ostend, Belgium). A Kolmogorov–Smirnov test was used to determine the normality of the data. Continuous data were calculated as mean ± standard deviation, and categorical variables were presented as the number of specimens and percentages. To compare the outcomes between the preoperative value and postoperative follow-up visits, one-way repeated measures ANOVA with a post-hoc test and the chi-square test were used. A *p*-value of < 0.05 was considered statistically significant.

## 3. Results

### 3.1. General characteristics and demographic data

A total of 20 patients diagnosed with unilateral hip disease were recruited in this study. Demographic data are presented in Table 1. The mean age of patients was 55.7 ± 15.1 years, and the mean body mass index (BMI) was 26.8 ± 4.4. Diagnostically, 12 patients (60%) had primary hip OA, 7 patients (35%) had avascular necrosis of the femoral head, and 1 patient (5%) had developmental dysplasia of the hip. All patients had a positive Trendelenburg sign (100%) with decreased hip abductor power and hip function. The mean hip abductor power and hip abductor recovery ratio were 96.3 ± 9.2 N and 50.7% ± 17.3%, respectively. The average operative time was 108 ± 14 min.

**Table 1:** Demographic data for the 20 patients with unilateral hip disease in this study.

	Value
Age, year	55.7 ± 15.1
Female gender	15 (75%)
BMI, kg/m^2^	26.8 ± 4.4
Disease onset, year	2.4 ± 1.2
Positive Trendelenburg test	20 (100%)
Affected hip VAS	7.9 ± 1.4
**Hip abductor power, newton**	
Affected side	96.3 ± 9.2
Control side	190.2 ± 54.6
Hip abductor recovery ratio, %	50.7 ± 17.3
Harris hip score	41.3 ± 9.0
WOMAC score	48.6 ± 11.1

### 3.2. Postoperative changes in hip abductor muscle power and hip abductor recovery ratio, and postoperative MRI

**Figure 3:**
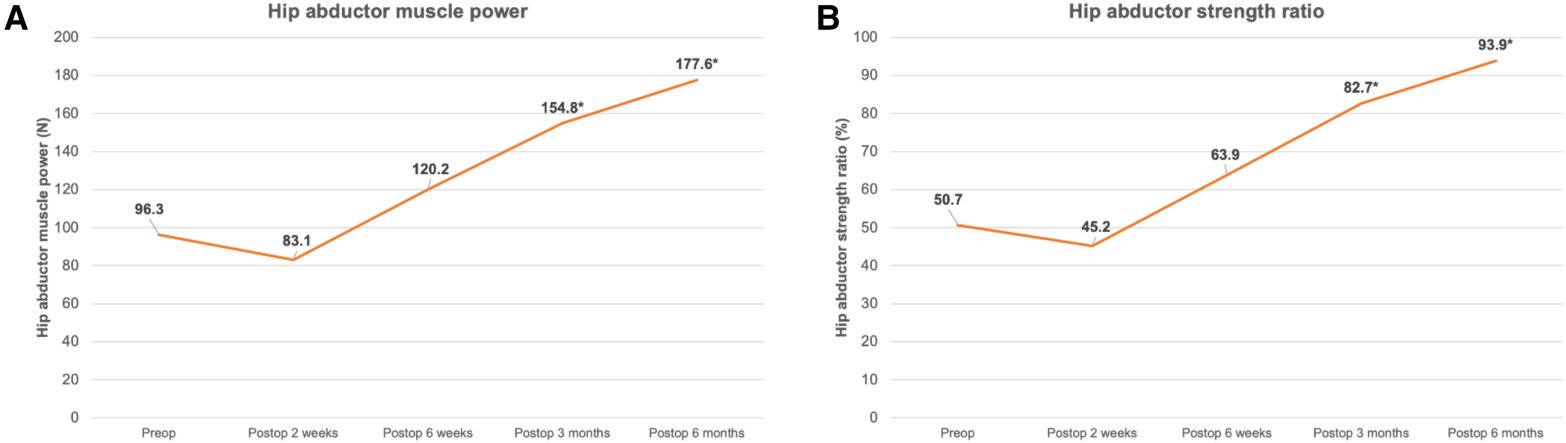
Postoperative changes in hip abductor muscle power (**A**) and hip abductor strength ratio (**B**). (*; significant difference compared with preoperative value, *p* < 0.05).

Figure 3 displays the postoperative changes in hip abductor muscle power and the hip abductor strength ratio. Table 2 shows the mean difference of the hip abductor strength ratio between the preoperative and postoperative periods, and the number of patients with a positive Trendelenburg sign at each follow-up visit.

**Table 2:** Mean difference of hip abductor strength ratio and the number of patients with positive trendelenburg sign at each follow-up visit.

Postoperative period	Hip abductor strength ratio^a^		Positive Trendelenburg sign^b^
	Mean difference	95% CI	
2 weeks	5.5 (4.3)	−8.1 to 19.2	20 (100%)
6 weeks	−13.2 (5.8)	−31.8 to 5.3	18 (90%)
3 months	−31.9 (5.9)*	−50.6 to −13.3*	12 (60%)*
6 months	−43.2 (5.0)*	−59.1 to −27.3*	4 (20%)*

^a^ Value presented as mean difference (standard error) between preoperative and postoperative values at each follow-up visit.

^b^ Vvalue presented as number of patients (percentage) having positive Trendelenburg sign at each follow-up visit.

*Significant difference compared to the preoperative value, *p* < 0.05.

Following THA using the AL approach, a non-significant decrease occurred in both hip abductor muscle parameters at 2 weeks postoperatively. However, both parameters were then improved and significantly increased after 3 months postoperatively compared with the preoperative values (*p* < 0.001).

Regarding the change in the Trendelenburg sign, a significant decrease also occurred in the number of patients with this sign after 3 months postoperatively compared to the preoperative value (*p* < 0.0001) (Table 2).

The MRI at the 9-month postoperative follow-up interval showed good healing without a gap in the muscle repair site for all patients. However, only 3 patients demonstrated minimal fibrosis and mild swelling of the anterior gluteus muscle.

### 3.3. Postoperative changes in VAS, Harris hip score, and WOMOAC score

**Figure 4:**
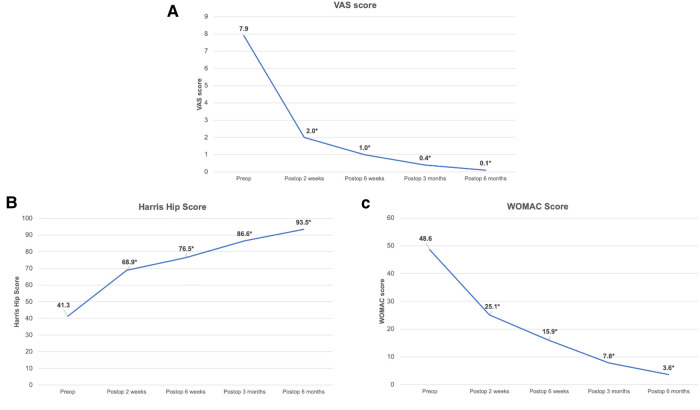
Postoperative changes in VAS (**A**), harris hip score (**B**), and WOMAC score (**C**). (*; significant difference compared with preoperative value with *p* < 0.05).

Figure 4 shows the postoperative change in the VAS, Harris hip score, and WOMAC score. Postoperatively, the pain score (VAS) and the functional scores (Harris hip score and WOMAC) significantly improved as early as 2 weeks postoperatively and showed continuous improvements throughout the 6-month follow-up period (*p* < 0.001).

## 4. Discussion

Abductor muscles of the hip play a major role in providing stability and mobility of the hips (22). The most common mechanisms leading to abductor muscle atrophy include degenerative or traumatic rupture of the tendon attachments, and iatrogenic injury during hip arthroplasty surgery (23, 24). The resulting insufficiency of abductor muscles not only causes pain and limits movement, but also leads to a typical gait abnormality known as Trendelenburg gait (25). In the AL approach for THA, the anterior one-third of the gluteus medius and gluteus minimus tendons must be detached from the greater trochanter of the femur and later repaired to ensure adequate exposure of the hip joint and allow for femoral dislocation. The main objective in our prospective cohort study was to evaluate the hip abductor muscle recovery, hip pain and function, and the healing of anterior hemimyotomy site of the gluteus medius in patients with unilateral end-stage hip OA undergoing THA with the AL approach.

The results in this study showed the hip abductor muscle power minimally and non-significantly decreased at 2 weeks postoperatively and then gradually increased later. At 3 months postoperatively, the hip abductor muscle power and the positive Trendelenburg sign had significantly improved compared with the preoperative period (Figure 2 and Table 2). However, despite the good healing of the anterior one-third hemimyotomy of the gluteus medius muscle based on MRI assessment, the hip abductor muscle did not demonstrate full recovery, as shown by the mean hip abductor strength ratio at 6 months postoperatively, which was 93.9% ± 2.2% compared to the contralateral normal side (Figure 2 and Table 2). These findings are comparable to the results from previous studies (17, 18, 26) and a previous meta-analysis by Ismailidis et al. in 2021 (27), all of which found that hip abductor muscle strength may gradually improve during the first 24 months post-THA and possibly without complete recovery. This outcome might be explained by the muscle atrophy and lower muscle volume in the hip OA patients (28, 29). Therefore, the results in the present study might imply that, in patients with unilateral end-stage hip OA who undergo THA with the AL approach, the hip abductor muscle could significantly improve without significant complications related to the muscle repair site but might not recover to the level equal to the contralateral normal hip.

Regarding the postoperative changes in pain and hip function, the present study showed that, after THA with the AL approach, the pain score significantly decreased as early as 2 weeks postoperatively and continuously decreased during the 6-month postoperative period (*p* < 0.05). The hip functional scores (Harris hip score and WOMAC score) also significantly improved postoperatively at 2 weeks and gradually improved over the entire follow-up period (Figure 3).

While the above results are insightful, the present study has some limitations. First, the sample size in this prospective cohort study is relatively small, and the follow-up period was only 9 months postoperatively due to patients' lack of motivation and the pandemic period of COVID-19. Nevertheless, several studies on AL approach also had the sample size estimation closed to the present study (between 16 and 26 patients) (30, 31). Also, previous studies showed that most of the cases with severe damage of nerves would still demonstrate the sign of denervation without recovery at 9 months (32–34). In addition, this study could still, however, provide the complete information related to the hip abductor muscle recovery and its related outcome through an MRI assessment in every case. Our results would therefore be helpful for encouraging surgeons to use the AL approach for THA. Second, we did not perform a comparative study to examine other approaches (e.g., direct anterior approach and posterior approach) because they were not the regular procedure at our institution. Finally, the hip abductor muscle power measurement in this study was performed using a handheld dynamometer rather than an isokinetic/isometric dynamometer as used in previous studies (17, 18). However, the handheld dynamometer has been widely accepted and used for measuring the muscle power in clinical studies, including hip arthroplasty studies (26, 35, 36). Previous studies using handheld dynamometers have also demonstrated good intra- and inter-observer reliability for isometric strength at the hip and knee, and moderate to high correlation values when compared to isometric dynamometry strength measures (37). Future studies comparing the benefits of the AL and DA approaches in patients with end-stage hip OA are needed to demonstrate the efficacy of AL approach in these specific patients.

In conclusion, the use of AL approach for THA in patients with end-stage hip OA effectively improved the hip clinical and functional outcomes as early as 2 weeks postoperatively without significant complications related to the hip abductor muscle. The hip abductor muscle recovery could be seen as early as 3 months postoperatively and then gradually increased during the 6-month postoperative period. However, based on our data, the postoperative hip abductor muscle power in these patients might not be fully recovered to match the normal side.

## Data Availability

The original contributions presented in the study are included in the article/Supplementary Material, further inquiries can be directed to the corresponding author.
